# Assortative Mating and the Reversal of Gender Inequality in Education in Europe: An Agent-Based Model

**DOI:** 10.1371/journal.pone.0127806

**Published:** 2015-06-03

**Authors:** André Grow, Jan Van Bavel

**Affiliations:** Centre for Sociological Research, University of Leuven, Leuven, Belgium; Texas Tech University Health Science Centers, UNITED STATES

## Abstract

While men have always received more education than women in the past, this gender imbalance in education has turned around in large parts of the world. In many countries, women now excel men in terms of participation and success in higher education. This implies that, for the first time in history, there are more highly educated women than men reaching the reproductive ages and looking for a partner. We develop an agent-based computational model that explicates the mechanisms that may have linked the reversal of gender inequality in education with observed changes in educational assortative mating. Our model builds on the notion that individuals search for spouses in a marriage market and evaluate potential candidates based on preferences. Based on insights from earlier research, we assume that men and women prefer partners with similar educational attainment and high earnings prospects, that women tend to prefer men who are somewhat older than themselves, and that men prefer women who are in their mid-twenties. We also incorporate the insight that the educational system structures meeting opportunities on the marriage market. We assess the explanatory power of our model with systematic computational experiments, in which we simulate marriage market dynamics in 12 European countries among individuals born between 1921 and 2012. In these experiments, we make use of realistic agent populations in terms of educational attainment and earnings prospects and validate model outcomes with data from the European Social Survey. We demonstrate that the observed changes in educational assortative mating can be explained without any change in male or female preferences. We argue that our model provides a useful computational laboratory to explore and quantify the implications of scenarios for the future.

## Introduction

The demography of educational attainment has important implications, not just for the individuals concerned, but also for long-term economic growth [1]. From this perspective, the dramatic changes in the relative educational attainment of men and women over the last decades represent a major social development. While participation in education beyond the primary level was expanding rapidly, higher education remained mostly a male domain until the 1970s. The gender gap has decreased since then in Europe, North America, and many other parts of the world, and by now, women largely excel men in terms of participation and success in higher education [2]. This implies that, for the first time in history, there are more highly educated women than men reaching the reproductive ages and looking for a partner.

Some scholars argue that this reversal of gender inequality in education will have important consequences for patterns of educational assortative mating (EAM) and ensuing family formation [3,4]. Yet, as Van Bavel [4] highlighted, shifts in the relative educational attainment of men and women are likely to have complex, non-linear effects on patterns of assortative mating, which makes it difficult to develop hypotheses about precisely *how* these shifts will affect EAM. In this paper, we address this problem by means of agent-based computational (ABC) modelling. We develop an agent-based model of European marriage markets that explicates the mechanisms that might have linked changes in educational attainment to recent changes in EAM.

**Fig 1:**
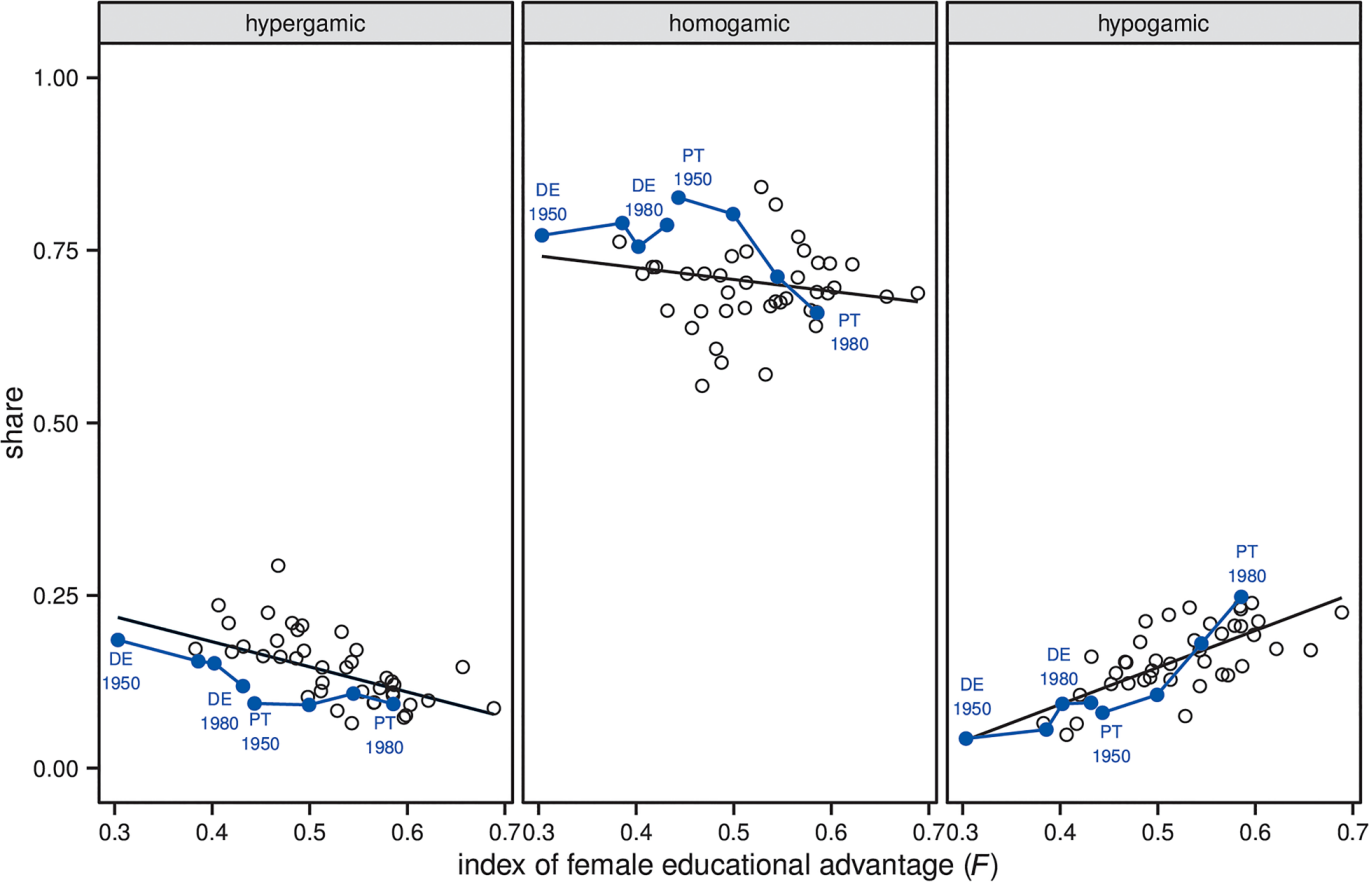
Relation between index of female educational advantage (*F*) and shares of hypergamic, homogamic, and hypogamic married couples. The selected countries are Belgium, Denmark, Finland, France, Germany, Greece, Ireland, The Netherlands, Portugal, Spain, Sweden, and United Kingdom. The selection is based on the data available for initializing the simulation model and for validating simulation outcomes. The figure shows for each country the four birth cohorts 1950 = (1940–1950], 1960 = (1950–1960], 1970 = (1960–1970], and 1980 = (1970–1980]. Data for calculating the index of female educational advantage *F* [3] come from the population reconstructions/projections by age, sex, and level of educational provided by the International Institute for Applied Systems Analysis/Vienna Institute for Demography, based on the age categories 30 to 34 and 35 to 39 years in 1980, 1990, 2000, and 2010. We used the global educational trend scenario for projections in 2010. The index is calculated as $F=\left[p_{f}^{3}\left(p_{m}^{1}+p_{m}^{2}\right)+p_{f}^{2}p_{m}^{1}\right]/\left[1-\left(p_{f}^{1}p_{m}^{1}+p_{f}^{2}p_{m}^{2}+p_{f}^{3}p_{m}^{3}\right)\right]$, where $p_{f}^{s}$ and $p_{m}^{s}$ refer to the shares of women (*f*) and men (*m*) in the ordered educational categories (*s*) 1 = ‘no education’/ ‘primary education’, 2 = ‘secondary education’, and 3 = ‘tertiary education’. The index equals 0 when every woman in the population holds a lower educational degree than any randomly selected man, 0.5 when men and women are equally educated, and 1 when every woman in the populations holds a higher educational degree than any randomly selected man. Data for calculating the shares of different types of married couples come from rounds 5 and 6 the European Social Survey. Weighting has been applied. Black lines are simple regression slopes fitted to the data.

First evidence shows that changes in the relative educational attainment of men and women have already affected patterns of assortative mating. The dominant pattern of EAM in 20^th^-century Europe has been homogamy combined with female hypergamy and male hypogamy. That is, women have tended to marry men who were at least as highly educated as themselves, whereas men have tended to marry women who were at most as highly educated as themselves. This pattern used to be compatible with the gender-specific distribution of educational attainment, but the larger the number of highly educated women became relative to the number of highly educated men, the less feasible the pattern became. Fig 1 illustrates how this change has affected patterns of EAM across 12 Western European countries. The figure suggests that over successive cohorts born between 1941 and 1980, increases in the female educational advantage were associated with a decrease in the shares of married couples in which the woman has the *lower* educational attainment (i.e. hypergamic couples) and with an increase in the shares of married couples in which the woman has the *higher* educational attainment (i.e. hypogamic couples). Also the shares of couples in which both partners have a *similar* educational attainment (i.e. homogamic couples) have decreased somewhat, but with more variation within countries than in the case of hypergamic couples [see 3 for a similar analysis that also includes non-European countries].

Earlier work that has documented the shift from hypergamy to hypogamy was unable to address the role played by preferences. Esteve and colleagues [3] speculate that the decrease in hypergamy to the benefit of hypogamy might reveal a change in the social preference for hypergamy, but could not draw any conclusions about the role played by changing preferences for mating patterns. This, they argue, is “a subject that should be addressed in future work” [3: 541].

In order to do that, we need a model that explicates the mechanisms that link preferences for potential mates and the gender-specific distribution of educational attainment with EAM. ABC modelling is particularly useful for developing such a model, because it enables us to deal with the complex, non-linear dynamics that the interactions among members of large populations can create [5–7]. One potentially important source of complexity in the relation between changes in the relative educational attainment of men and women and EAM is the fact that individuals evaluate potential mates based on preferences for multiple characteristics. These preferences can contribute differently to patterns of EAM, depending on the structure of the marriage market. ABC modelling enables us to address this complexity by assessing the relation between preferences and the structure of the marriage market with analytical rigor [8,9]. Furthermore, ABC modelling helps us to avoid some of the limitations that have been criticized in earlier mathematical marriage market models. In particular, earlier models have been criticized for their often static nature [10] and for sometimes lacking a firm grounding in theories of individual behaviour [11]. With ABC modelling, we can specify the rules for individual behaviour based on existing theory and study their interplay in a dynamic system [7].

This paper presents an agent-based model of union formation and educational assortative mating based on well-established principles of individual mate search. In developing our model, we build on earlier empirical research that has studied the interplay between marriage market constraints and partner preferences in generating patterns of assortative mating [e.g., 12–14] and on earlier simulation work that has centred on human mate search [e.g., 15,16]. Our focus is on partner preferences in terms of age, education, and earnings prospects, because these attributes are commonly ranked among the most important criteria for partner selection [cf. 14,17] and have been assumed to play a central role in linking the gender-specific distribution of educational attainment with EAM [4]. Additionally, we take into account that the educational system might have a structuring effect on meeting opportunities on the marriage market and thereby might additionally affect patterns of EAM [18–20].

Advocates of ABC modelling in population research increasingly highlight the benefits of backing simulation models with demographic data [9,21]. To generate realistic agent populations in terms of educational attainment, we use data provided by the International Institute for Applied Systems Analysis/Vienna Institute for Demography (IIASA/VID) [22,23]. The IIASA/VID data provide reconstructions (from 1970 until 2000) and projections (from 2005 until 2050) of the distribution of educational attainment in 5-year age groups for a large number of countries. To generate realistic agent populations in terms of earnings prospects, we pair the IIASA/VID data with information from the European Community Household Panel (ECHP) on the relative earnings prospects of men and women contingent on their educational attainment. For calibrating model parameters and for validating model outcomes, we use data from rounds 5 and 6 of the European Social Survey (ESS), collected in 2010 and 2012 [24,25], focusing on countries for which data was available in both rounds. The combination of the three data sources enabled us to simulate mate search between 1921 and 2012 in 12 Western European countries, under realistic demographic conditions.

Given that our model proves to be able to generate realistic patterns of EAM and their change over time, this paper makes at least three contributions to the literature on human mating behaviour. First, we show that the age-old pattern of hypergamy (‘women marrying up’ in terms of education) is compatible with a preference for homogamy and can be explained without any recourse to an explicit preference for hypergamy. Second, we demonstrate that the shift from hypergamy to hypogamy (‘women marrying down’) can be explained without the need to assume shifting preferences over time. Third, our model, tried and tested with historical empirical data, can be used as a virtual laboratory to explore potential ‘what-if’ scenarios for the future. For example, recent literature on human mating behaviour suggests that gender specific mate preferences may be changing in the years to come [26]. Our model allows quantifying the implications of such and alternative scenarios for assortative mating, which are bound to have implications for human reproduction [4].

In the remainder, we first discuss existing research on human mate search and outline how the mechanisms that earlier studies have described might link the gender-specific distribution of educational attainment with EAM. Subsequently, we develop the formal model and submit it to systematic computational experiments. We close the paper with a discussion of results and provide an outlook for future research. The model code, the data for our analysis, and the syntax of our analyses can be obtained from http://dx.doi.org/10.7910/DVN/HPUQ1Y.

## Theoretical Background

Scholars interested in explaining population level mating patterns commonly employ the notion of the marriage market [14,20,26]. This notion holds that individuals “search for partners in a market in which people have preferences for mates but face constrained opportunities” [26: 452]. Constrained opportunities, in turn, can affect their aspirations during partner search [27,28]. We follow this tradition here and employ the notion of the marriage market for modelling the link between the gender-specific distribution of educational attainment and EAM. In this section, we therefore first specify the *preference structures* that govern mate selection in our model, explicate the *constraints* that the marriage market imposes on mate search, and discuss how *aspirations change* in response to individuals’ position on the marriage market. Subsequently, we discuss how these factors might interact in generating the observed relation between changes in the relative educational attainment of men and women and patterns of EAM.

### Preferences

People possess a number of attributes that can make them more or less desirable on the marriage market [for surveys of such attributes see 29, 30]. Age, education, and earnings prospects have received particular attention in earlier research and have been found to play a central role during mate search. Existing evidence suggests that both men and women tend to prefer spouses who are similar to them in educational attainment and who have high earnings prospects [29–36]. When it comes to age, the available evidence indicates that women tend to prefer men who are somewhat older than themselves, but typically not more than 10 years. Men tend to prefer women who are in their mid-20s, regardless of their own age [37–39]. This implies that men who have not reached their mid-twenties yet tend to be attracted to somewhat older women [cf. 38].

Preferences for spouses with similar educational attainment and high earnings prospects have been assumed to derive from the resources that these attributes are linked to [14,31]. Education, on the one hand, is indicative of the *cultural resources* that individuals have at their disposal [32]. Cultural resources encompass “values and behaviours, such as child-rearing values, political attitudes, cultural literacy, taste in art and music, and styles of speech” [31: 426]. Within couples, similarity in such values and behaviours can lead to mutual reinforcement of world views, create feelings of social confirmation, and facilitate the organization of joint activities. People therefore tend to prefer spouses with similar cultural resources [34]. Earnings prospects, on the other hand, are a proxy of the long-term *economic resources* that individuals will have at their disposal [13]. Within couples, economic resources are typically shared. Thus, all else equal, people prefer partners with higher earnings prospects in order to maximize their long-term economic well-being. In the traditional male breadwinner model, women have attached particular importance to the earnings potential of their spouses due to their own lower involvement in the labour market. This rendered the income of the husband often more important for the financial status of the family than the income of the wife. Changes in female labour force participation and changing gender roles, however, have potentially led the importance that man and women attach to the earnings prospects of their partners to become more similar [13,40].

Explanations of gendered age preferences have largely been developed within two different theoretical frameworks. Explanations based on socio-cultural learning in sociology [37,39,41] argue that gendered age preferences are based on a double standard of aging in society and on segregated gender roles. According to this explanation, women are judged more by their physical attractiveness than men and the ideal of female beauty “is best exemplified by women about age 20 years” [37: 800]. Men therefore have a preference for women who are in their early twenties, regardless of their own age. Women’s preference for somewhat older men has been assumed to derive from the fact that the status of a family used to derive mainly from the income of the husband. To the extent that the financial prospects of younger men are less certain than that of older men, women therefore tend to prefer somewhat older men [41].

Explanations based on evolutionary biology [36,38,42] assume that selection pressures in the ancestral environment of humans have hard-wired gendered age preferences. Female fecundity peaks around their mid-20s and then decreases until menopause sets in the 50s; mating with women who are in their twenties therefore held fitness benefits for men. Male fecundity varies less with biological age and this renders men’s age less relevant for generating offspring [36,43]. Yet, very young men were often less able to defend offspring and very old men face increasing physical frailty and increasing risks of morality, which might prevent them from caring for their offspring until it reaches maturity. In both cases, the fitness value of a relation would be reduced; evolutionary processes have therefore hard-wired women to prefer men who are somewhat older, but not too much older [38].

The alternative explanations that sociology and evolutionary biology offer for gendered age preferences are potentially complementary and both imply that the market value of women deteriorates faster with age than the market value of men

### Marriage market constraints

The preference structures that we have outlined describe ideals that men and women tend to have in mind when looking for a spouse. The extent to which these ideals can be realized depends on the composition of the marriage market and on the way in which meeting opportunities are structured [12–14,27,44].

The prospects of finding an ‘ideal match’ depends on the supply of, and demand for, individuals with the desired attributes [44]. Consider first the effect of supply. The chance that a highly educated man will find a similarly educated partner, for example, depends on the availability of highly educated women in the population. If the number of highly educated men is higher than the number of highly educated women, it is impossible that every highly educated man will be able to find an ideal match in terms of education. Consider next the effect of demand. If there is a relative shortage of highly educated women, the earnings prospects among highly educated men will lead to an inequality in the chances of these men being able to find an ideal match. The reason is that women tend to value men with high earnings prospects over men with low earnings prospects. The former group of men will thus be better able to compete for highly educated women than the latter group.

Given the composition of the marriage market, scholars have highlighted that mating crucially depends on meeting opportunities and such opportunities are often not distributed randomly [12,45]. Instead, they tend to centre on small functional places, such as schools, neighbourhoods, and workplaces, and these ‘local marriage markets’ are often socially segregated [14]. The school system as a local marriage market has received particular attention in existing research on EAM [18,20,45–47]. The reason is that many important mating decisions take place during the transition from adolescence to adulthood [20]. During this period, individuals typically attend school and the people they meet there tend to account for large shares of their social networks [48]. This increases the likelihood that people meet their first spouse at school. Furthermore, the longer individuals stay in the educational system, the more homogenous their school environment becomes in terms of ultimate educational attainment [18]. At the secondary level, for example, classes consist of both individuals who leave school after obtaining a secondary degree and individuals who will move on to the tertiary level. At the tertiary level, by contrast, classes mostly consist of individuals who will obtain a tertiary degree. As a consequence, at higher levels of the educational system, there is an increased chance that spouses who met at school are educationally homogamic. Due to this structuring effect, the educational system has often been assumed to be a driving force behind traditionally high levels of educational homogamy, next to preferences and the overall composition of the population [18–20].

### Changing aspirations

The search for a spouse is a search under uncertainty: people typically have imperfect information on the composition of the marriage market and therefore do not know whether and when they will find an ideal match [13]. The less favourable the marriage market conditions are, the more difficult it becomes to find such a match and the longer the search might take. The longer people are already looking for a partner, the more risky it becomes to pass up on potential spouses in the hope of finding a better match in the future. This risk derives partly from the fact that individuals’ position on the marriage market tends to worsen with increasing age. For women, male preferences imply that their market value decreases once they are past their mid-twenties [39]. This makes it increasingly difficult for them to find a partner of about the same age, once they have passed the age of 30. It also becomes more difficult for men to find an ideal match as they become older. The reason is that women tend to prefer men around their own age. This makes it increasingly difficult for men to find a woman who is in her mid-twenties and who is also willing to take them as a partner as they become older themselves.

Existing research suggests that people adapt to these changes in their position on the marriage market by adjusting their aspirations for prospective spouses. That is, people tend to be very selective in their early twenties, when their position on the marriage market is most conducive to finding a partner. As they become older without having a partner, they cast an increasingly wider net and become increasingly willing to accept partners who are less than an ideal match in terms of their ideals [27,28].

### Linking changes in relative educational attainment with EAM

How might the preferences and marriage market constraints that we have discussed link the gender-specific distribution of educational attainment with EAM? Evidently, the distribution of educational attainment in a given population will affect the likelihood with which individuals can find a similarly educated partner. The more similar men and women are in their average educational attainment, the more likely it is that they will find similarly educated partners, and the higher the level of homogamy will be. The structuring effect that the educational system has on meeting opportunities potentially strengthens this association [4]. Yet, if men are on average more highly educated than women, there will be a shortage of similarly educated men among medium and lower educated women. If women desire marriage and lower their aspirations to avoid foregoing marriage, such an imbalance is likely to increase hypergamy. Conversely, if women are on average more highly educated than men, hypogamy is likely to increase.

Van Bavel [4] highlighted that individuals’ preferences in terms of age and earnings prospects might intervene in the effect that the availability of similarly educated spouses has on assortative mating. Consider first individuals’ preferences related to age. Highly educated women born in more recent cohorts are likely to face a shortage of similarly educated men who are about their own age. In earlier birth cohorts, by contrast, such men are relatively abundant. For women, one way to find a similarly educated spouse might therefore be to look for partners who are much older than themselves. If they would indeed opt for much older partners, we might expect that the level of educational homogamy changes comparatively little, even if there is a shortage of highly educated men in recent cohorts. Yet, there is a limit to women’s willingness to accept older partners. This limit reduces the feasibility of looking for similarly educated spouses in older cohorts and this potentially strengthens the effect that changes in the relative educational attainment of men and women have on EAM.

**Fig 2:**
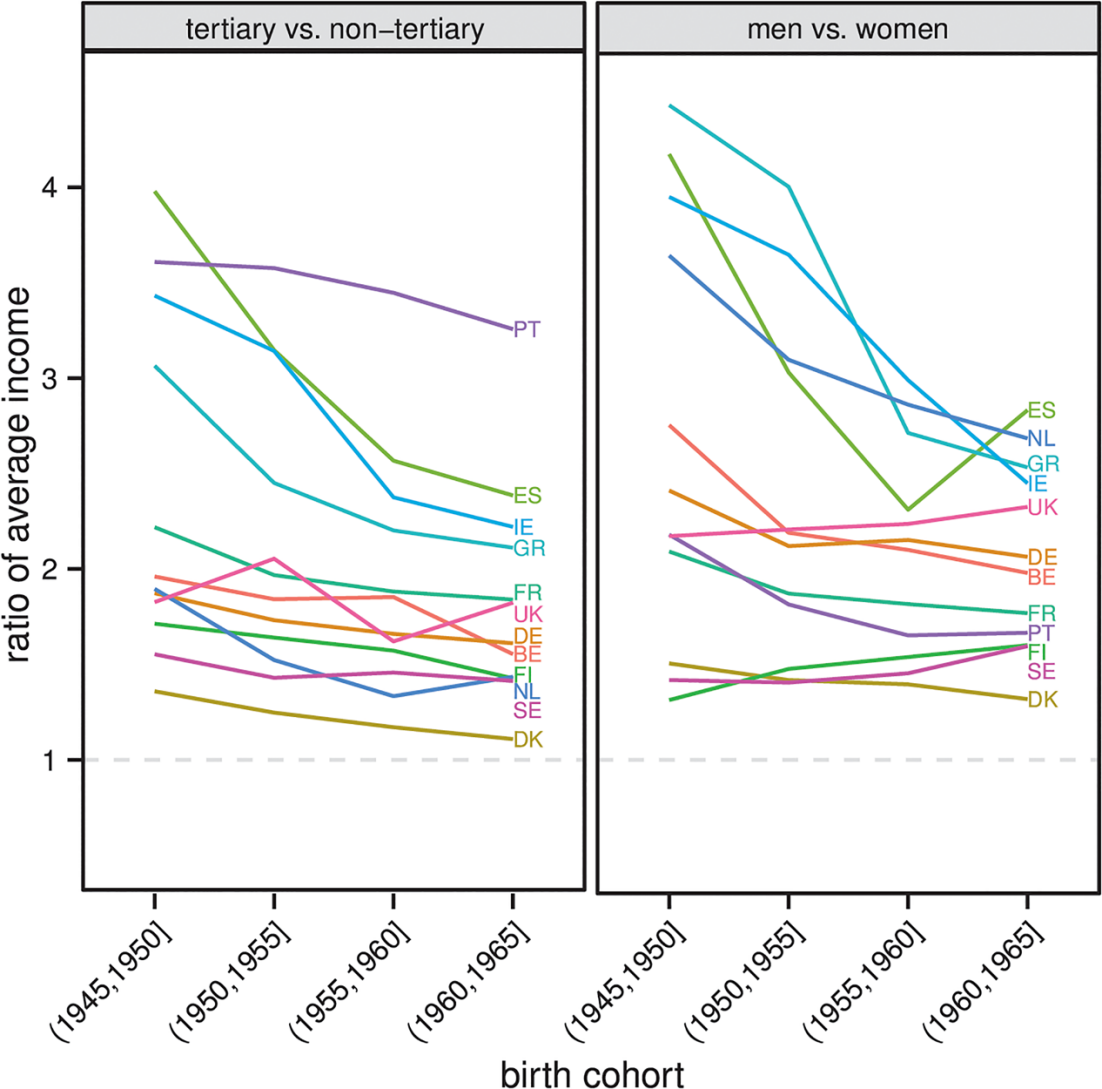
Income comparison based on education and gender. The selected countries are Belgium, Denmark, Finland, France, Germany, Greece, Ireland, Netherlands, Portugal, Spain, Sweden, and United Kingdom. The selection is based on the data available for initializing the simulation model and for validating simulation outcomes. Data for calculating the relative average income of men and women come from the ECHP, waves 1 to 8. Income refers to the annual net income from work in the year prior to the survey among respondents who were not retired or self-employed at any point during the income reference period. Information on employment status was not available for The Netherlands and Sweden; in these countries all cases were retained. The focus is on respondents who were 36 to 50 years old during the income reference period. Weighting has been applied.

Consider next individuals’ preferences related to earnings prospects. Education is an important determinant of economic resources, but women tend to earn less than men. Fig 2 illustrates these earnings differences for the same 12 European countries shown in Fig 1, based on data taken from the ECHP. Existing research suggests that earnings differences between men and women partly derive from factors such as the traditionally weaker attachment of women to the labour market, differences in the earning potential of the study fields that men and women tend to choose, and gender discrimination [49,50]. Regardless of the causes of the difference in the earnings prospects of men and women, the general correlation between education and income might contribute to both educational homogamy and heterogamy, depending on the structure of the marriage market. When the numbers of highly educated men and women are balanced, highly educated people can easily find similarly educated partners and the high earnings prospects of these individuals adds to the attractiveness of marrying within their own educational group. Yet, when there is a relative shortage of highly educated men or women, the correlation between earnings prospects and education might contribute to heterogamy. The reason is that those highly educated individuals who fail to find a similarly educated partner are very attractive on the marriage market, even for lower educated individuals. This makes it easier for them to find a partner outside their own educational group.

The foregoing intuitions illustrate how the preferences that we consider here might interact with the structure of the marriage market in generating observed patterns of EAM. However, due to the complexity of these interactions, our intuitions might not be correct and it therefore remains unclear to what extend the mechanism that we have described can explain observed changes in EAM. To deal with this problem of complexity, we translated our theory into an agent-based model that enables us to study the interplay between preferences and marriage market constraints in generating patterns of EAM with analytical rigor.

## An Agent-Based Marriage Market Model

Earlier agent-based models of human mate search have focused on population level patterns of age at first marriage [15,16,51–53], divorce [54], and the formation of (multiple) sexual relationships [55]. Our model is, to the best of our knowledge, the first to focus on patterns of EAM.

In our model, agents look for spouses who are similar to them in terms of educational attainment and who have high earnings prospects. Additionally, female agents look for spouses who are somewhat older than themselves, whereas male agents look for spouses who are in their mid-twenties. The closer a member *j* of the opposite sex comes to agent *i*’s ideals in terms of education, earnings prospects, and age, the more attractive *j* becomes as a spouse for *i*. In line with earlier modelling work, we express this attractiveness in a single number, the *mate value* [e.g., 16]. Based on the empirical insight that individuals’ position on the marriage market tends to deteriorate with age, which typically makes them cast a ‘wider net’ [27,28], we assume that younger agents are more selective than older agents in choosing a partner based on his/her mate value. Similar effects of age have been included in a number of earlier models [e.g., 15,16,51,54].

We model the process of mate search over the entire life course. From the moment agents reach a marriageable age, they actively search for a spouse on the marriage market and randomly encounter members of the opposite sex. We allow for the possibility that a given agent *i* meets another agent *j* can be structured by the educational system. That is, agents who are currently attending the same educational level might be more likely to meet each other than agents who attend different educational levels. Whenever two agents meet, they need to decide whether they want to start dating, which can, in the long run, lead to marriage. The higher the mate values that two agents perceive in each other, the more likely dating and marriage become.

Agents can continue to meet members of the opposite sex even when they are currently in a relationship, with the goal to find somebody who has an even higher mate value than their current partner. If they find such an alternative, they might leave, or divorce, their current partner, depending on whether they are currently dating or married. However, in line with earlier modelling work, we assume that agents become less likely to actively search for better alternatives and to break up with their current partner, the longer they have been in their current relationship [e.g., 15,16]. This implements the notion that couples tend to build up intimacy and become more involved over time.

In the remainder of this section, we describe the different elements of the model in detail. Tables 1 and 2 provide overviews of the symbols that we introduce in this section. We implemented the model in NetLogo, version 5.1 [56].

**Table 1:** Overview of model parameters.

Parameters	Description	Values/ranges used in experiments
*A*_*marr*_	Age at which agents become marriageable	160
*S*_*max*_	Maximal education that agents can attain	4
*Y*_*max*_	Maximal earnings prospects that agents can have	5
*A*_*max*_	Maximal age that agents can reach	800
$w_{s}^{m},w_{s}^{f}$	Importance that male and female agents attach to similar education of partners	0 ≤ *w*_*s*_ ≤ 2
$w_{y}^{m},w_{y}^{f}$	Importance that male and female agents attach to high earnings prospects of partners	0 ≤ *w*_*y*_ ≤ 2
$w_{a}^{m},w_{a}^{f}$	Importance that male and female agents attach to the age of partners	0 ≤ *w*_*a*_ ≤ 20
*ß* ^*m*^, *ß* ^*f*^	Commitment factor for male and female agents	.015,. 015
*σ* ^*m*^, *σ* ^*f*^	Age pressure factor for male and female agents	.0015,. 0030
*δ*	Structuring effects of the educational system	0 ≤ *δ* ≤ 1
*d* ^*m*^, *d* ^*f*^	Maximal probability of death at age *A*_*max*_	.1,. 1
$w_{d}^{m},w_{d}^{f}$	Parameters that govern the shape of the age specific probability of death	6, 6

**Table 2:** Overview of agent level variables.

Variables	Description	Values/ranges used in experiments
*g*_*i*_	Gender	∈ {1, 2} ^a^
*a*_*i*_	Age	0 ≤ *a*_*i*_ ≤ *A*_*max*_
*α*_*i*_	Ideal age that agent *i* prefers in a partner	0 ≤ *α*_*i*_ ≤ *A*_*max*_
*s*_*i*_	Highest educational level that the agent will ever attain	∈ {1, 2, 3, 4} ^b^
*r*_*i*_	School enrolment status	∈ {1, 2, 3, 4, 5} ^c^
*y*_*i*_	Earnings prospects	∈ {1, 2, 3, 4, 5} ^d^
*l*_*i*_	Relationship status	∈ {1, 2, 3} ^e^
*c*_*i*_	Duration of current relation	0 ≤ *c*_*i*_ ≤ *A*_*max*_
*v*_*ij*_	Mate value that agent *i* perceives in agent *j*	0 ≤ *v*_*ij*_ ≤ 1

^a^1 = 'male' and 2 = 'female'

^b^1 = 'no education', 2 = 'primary education', 3 = 'secondary education', and 4 = 'tertiary education'

^c^1 = ‘not in the educational system yet’, 2 = ‘in primary education’, 3 = ‘in secondary education’, 4 = ‘in tertiary education’, and 5 = ‘finished education’

^d^1 = 'less than 20% of the income of the top earning category', 2 = '20% or more and less than 40%', …, and 5 = '80% or more'

^e^1 = 'single', 2 = 'dating', 3 = 'married'

### Agent characteristics

Our model proceeds in discreet time steps and all time related elements are expressed in these steps. Ten time steps represent one simulation year. Each agent can be described by its gender *g*_*i*_ (1 = ‘male’ or 2 = ‘female’), age *a*_*i*_ (measured in time steps), the highest educational level that it will ever attain *s*_*i*_ (1 = ‘no education’, 2 = ‘primary education’, 3 = secondary education’, or 4 = ‘tertiary education’), school enrolment status *r*_*i*_ (1 = ‘not in the educational system yet’, 2 = ‘in primary education’, 3 = ‘in secondary education’, 4 = ‘in tertiary education’, or 5 = ‘finished education’), relationship status *l*_*i*_ (1 = ‘single’, 2 = ‘dating’, or 3 = ‘married’), the time it is already in a relationship with its current partner *c*_*i*_ (measured in time steps), and the ideal age it prefers in a partner *α*_*i*_. Additionally, agents can be described by their earnings prospects *y*_*i*_, expressed in five categories (i.e. 1, 2, …, or 5). Higher values on *y*_*i*_ represent higher earnings prospects; for details regarding these categories see S1 Appendix. The characteristics *g*_*i*_, *s*_*i*_, *α*_*i*_, and *y*_*i*_ are assigned when an agent is born (i.e. enters the simulation) and remain stable over the course of a given simulation run. The characteristics *a*_*i*_, *r*_*i*_, and *l*_*i*_ can change over the course of a simulation run and are updated in each simulation step.

The values of *a*_*i*_ and *s*_*i*_ together determine agents’ school enrolment status *r*_*i*_. That is, whenever agents reach the age at which they drop out of the educational system or make the transition to the next educational level, the value of *r*_*i*_ is updated accordingly. In our model, primary education begins at the age of 6 (*a*_*i*_ = 60), secondary education at the age of 10 (*a*_*i*_ = 100), and tertiary education at the age of 19 (*a*_*i*_ = 190). All agents transfer from one educational level to the next, unless they have dropped out of the educational system. Agents who attain no education (*s*_*i*_ = 1) never enter the educational system and thus are assigned the state ‘finished education’ already at the age of 6 (*a*_*i*_ = 60). Agents who attain primary education as their highest degree (*s*_*i*_ = 2) leave school at the age of 16 (*a*_*i*_ = 160). This implements the fact that a minimum number of years are typically compulsory, so that pupils are required to participate in secondary education for some time, even if it is not completed successfully. Agents who attain a secondary degree as their highest degree (*s*_*i*_ = 3) leave school at the age of 19 (*a*_*i*_ = 190). Agents who attain a tertiary degree as their highest degree (*s*_*i*_ = 4) leave school at the age of 24 (*a*_*i*_ = 240).

### Partner preferences

Agents reach a marriageable age at 16 (*A*_*marr*_ = 160). From this moment on they try to find a spouse who has also reached a marriageable age and has a high mate value *v*_*ij*_. We calculate this value as

$$v_{ij}={\left(\frac{S_{\text{max}}-\left|s_{i}-s_{j}\right|}{S_{\text{max}}}\right)}^{w_{s}}{\left(\frac{y_{j}}{Y_{\text{max}}}\right)}^{w_{y}}{\left(\frac{A_{\text{max}}-\left|\alpha_{i}-a_{j}\right|}{A_{\text{max}}}\right)}^{w_{a}}.$$ (1)

In Eq (1), *S*_*max*_, *Y*_*max*_, and *A*_*max*_ are the maximal education, earnings prospects, and age that agents can reach; the parameters *w*_*s*_, *w*_*y*_, and *w*_*a*_ govern how much deviations from the ideals that agents hold for a potential spouse lower the value of *v*_*ij*_. The value of *v*_*ij*_ can vary continuously from 0 to 1. From agent *i*’s point of view, the more similar *i* and *j* are in their ultimate educational attainment, the higher the earnings prospects of *j*, and the closer *j* is to the age that *i* desires in a partner, the closer *v*_*ij*_ comes to 1. Deviations from these ideals decrease the value of *v*_*ij*_, and this decrease is stronger at higher values of *w*_*s*_, *w*_*y*_, and *w*_*a*_ respectively. We allow these parameters to differ between male and female agents, so that it is possible that $w_{s}^{m}\neq w_{s}^{f}$, $w_{y}^{m}\neq w_{y}^{f}$, and $w_{a}^{m}\neq w_{a}^{f}$. This implements the notion that men and women might differ in the importance they attach to each of the three dimensions [30].

Note that *s*_*i*_ and *y*_*i*_ remain stable over agents’ life course. Thus when agents assess each other’s mate value, we assume that they anticipate the highest educational level a potential partner will ever attain and how high his/her life-time earnings prospects will be.

### Partner search and effects of the educational system

Agents might try to find a spouse on the marriage market, but their inclination to do so decreases the longer they are in a relationship with their current partner (either in the form of dating or marriage). More formally, the probability that agent *i* will actively seek out other agents in the current time step (Pr(*i seek*)) is determined by

$$\text{Pr}(i\;seek)=e^{-\left(c_{i}\beta \right)}\;.$$ (2)

In Eq (2), *β* is an exogenous factor that determines the effect that the time that agent *i* is already in a relationship with his/her current partner (*c*_*i*_) has on the probability that *i* will try to meet somebody. From here on, we refer to *β* also as the ‘commitment factor’. When *β* > 0, the longer a given agent is already in his/her current relationship (i.e. the larger *c*_*i*_ becomes), the less likely the agent becomes to actively search out members of the opposite sex. This decrease accelerates at higher values of *β*. Note that for single agents, *c*_*i*_ is always 0; the probability that they will actively search out somebody is therefore always 1. We allow that the parameter *β* can differ between male and female agents, so that it is possible that *β* ^*m*^ ≠ *β* ^*f*^.

If it has been determined that agent *i* will actively seek out somebody in the current time step (with the probability determined in Eq 2), a member *j* of the opposite sex is selected at random from the marriage market, and this selection is affected by the structuring effect of the educational system. More specifically, *j* can be selected from two sets of agents: agents who have the same school enrolment status *r*_*i*_ as *i* (i.e. *r*_*i*_ = *r*_*j*_), and agents who have a different school enrolment status *r*_*i*_ than *i* (i.e. *r*_*i*_ ≠ *r*_*j*_). The probability with which *j* is selected from each of the two sets is determined by the ‘structuring parameter’ *δ* (0 ≤ *δ* ≤ 1), so that

$$\text{Pr}(r_{i}=r_{j})=\delta$$ (3A)

and

$$\text{Pr}(r_{i}\neq r_{j})=1-\delta \;.$$ (3B)

Thus, the closer the value of *δ* is to 1, the more likely agents are to meet somebody with the *same* school enrolment status; the closer value *δ* is to 0, the more likely they are to meet somebody with a *different* school enrolment status. When *δ* is. 5, agents are as likely to meet somebody with the same school enrolment status, as they are to meet somebody with a different school enrolment status.

### Dating, marriage, and age pressure

Whenever two agents meet, they need to decide whether they want to start dating. Single agents consider anybody they meet as a possible date. Agents who are currently in a relationship (either in the form of dating or marriage), by contrast, only consider those as a possible date whose mate value is higher than the mate value of their current partner (i.e. when $v_{ij}^{alternative}>v_{ij}^{partner}$). When both agents are willing to date, they enter a relationship and form a couple, for which they leave/divorce possible current partners. The probability that a given agent is willing to date another agent *j* (Pr(*i date j*)) is determined by

$$\text{Pr}(i\;date\;j)=\left(1-e^{-\left(a_{i}v_{ij}\sigma \right)}\right)e^{-\left(c_{i}\beta \right)}\;.$$ (4)

In Eq (4), *σ* is an exogenous factor that governs the pressure to find a partner that agents experience as they become older. From here on, we refer to *σ* also as the ‘age pressure factor’. The first term of Eq (4) implies that if *σ* > 0, the likelihood that agent *i* is willing to date another agent *j* typically increases with *j*’s mate value. However, the older agents become, the more willing they become to date anybody; when *σ* = 1, agents of any age are willing to date *any* other member of the opposite sex, regardless of the value of *v*_*ij*_. Yet, the second term of Eq (4) implies that this willingness can be attenuated when agents are currently in a relationship. Note again that for single agents, *c*_*i*_ is always equal to zero. For such agents, the second term of Eq (4) is therefore always equal to 1. As a consequence, all that matters for their willingness to start dating somebody is their own age (*a*_*i*_) and the mate value of the potential partner (*v*_*ij*_), in combination with the age pressure factor (*σ*). By contrast, for agents who currently have a partner, the value of Pr(*i date j*) is attenuated by the time they are already in the relationship (*c*_*i*_), in combination with the commitment factor (*β*). We allow that the parameter *σ* can differ between male and female agents, so that it is possible that *σ* ^*m*^ ≠ *σ* ^*f*^.

The longer a given agent is already dating his/her current partner, the more willing he/she becomes to marry and therefore to propose marriage/to accept a marriage proposal from his/her partner. From the moment agent *i* (or *j*) proposes marriage to his/her partner *j* (*i*), the proposal remains intact until *j* (*i*) agrees to marry, or until one of them terminates the relationship or dies. They get married at the moment both agree to marry. We model the probability that agent *i* proposes to/is willing to accept a proposal from his/her current partner *j* (Pr(*i marry j*)) by

$$\text{Pr}(i\;marry\;j)=\left(1-e^{-\left(a_{i}v_{ij}\sigma \right)}\right)\left(1-e^{-\left(c_{i}\beta \right)}\right)\;.$$ (5)

Eq (5) holds that older agents are generally more likely to propose marriage/accept proposals, and this likelihood increases with the mate value of their partner (*v*_*ij*_), the length of their relationship (*c*_*i*_), the age pressure (*σ*), and the commitment factor (*β*).

Our model allows for divorce in a simple way, mirroring the general rules for starting a relationship. That is, agent *i* potentially leaves his/her current partner *j* when he/she finds an alternative *k* that has a higher mate value than *j*. All else being the same, the probability that *i* tries to start a new relation with *k* (and therefore leaves *j*, if *k* is also willing to start a relation with *i*) is the same when *i* and *j* are dating or when they are married. Furthermore, agents only break up with or divorce their current partner when at least one of them finds a better alternative.

### Death and reproduction

Given that we are interested in modelling mate search over several cohorts, we need to make auxiliary assumptions about death and reproduction of agents. Thus, in each simulation step, there is a chance that a given agent dies and this chance increases convexly with its age. We model this by

$$\text{Pr}(i\;death)=.1d{\left(\frac{A_{\text{max}}-\left|A_{\text{max}}-a_{i}\right|}{A_{\text{max}}}\right)}^{w_{d}}\;,$$ (6)

where *d* is a factor that determines the maximum probability of death at the age of 80 (*a*_*i*_ = 800) and *w*_*d*_ governs the shape of the function. The scaling factor. 1 takes into account that each simulation year consists of ten time steps in which a given agent can die. Agents who have not died by the age of 80 (i.e. *A*_*max*_ = 800) yet are automatically removed from the population. We allow that the parameters *d* and *w*_*d*_ can differ between male and female agents, so that it is possible that *d* ^*m*^ ≠ *d* ^*f*^ and $w_{d}^{m}\neq w_{d}^{f}$.

Each agent who leaves the population (either because it dies or because it is removed at the age of 80) is replaced by a new agent with the same gender who starts its life with *a*_*i*_ = 0.

## Computational Experiments and Results

Can our model explain the link between the changes in the gender-specific distribution of educational attainment and observed changes in patterns of EAM? To what extent do preferences for education, earnings prospects, and age contribute to this link? And what does the structuring effect of the educational system add to this? To answer these questions, we conducted systematic computational experiments in which we simulated mate search processes across European countries, under realistic marriage market conditions in terms of the gender-specific distribution of educational attainment and earnings prospects. To create realistic marriage market conditions, we paired data from the IIASA/VID with data from the ECHP. This enabled us to simulate mate search among individuals born between 1921 and 2012, across 12 Western European countries. S1 Appendix discusses the way in which we paired the IIASA/VID data with data from the ECHP and how we used this data to initialize agent cohorts during simulation runs.

We compared the outcomes of our experiments with patterns of assortative mating observed among married couples in rounds 5 and 6 of the ESS. The IIASA/VID, ECHP, and ESS measure education based on the International Standard Classification of Education (ISCED), but some categories are combined in the IIASA/VID data. We harmonized the measurement of education in all three data sources so that education is measured as 1 = ‘no education’, 2 = ‘primary education’, 3 = ‘secondary education’, and 4 = ‘tertiary education’, which is the same as in our simulation model. Note that the observations for category 1 were very low in all data sources. We therefore combined the categories 1 and 2 when calculating outcomes.

In this section, we report the outcomes of two sets of computational experiments. The first set aimed at assessing whether our model is able to explain observed changes in EAM. The second set aimed at assessing how much the different processes that our model implements might have contributed to this link. The focal outcomes in all our analyses were shares of hypergamic, homogamic, and hypogamic married couples in a given country, among the members of the four birth cohorts (1940–1950], (1950–1960], (1960–1970]), and (1970–1980]). The selection of these cohorts was based on the fact that at the time the data for the ESS was collected (in 2010 and 2012), members of these cohorts were old enough (i.e. 30 years at minimum) to have already attained their highest educational degree, but not so old (i.e. 71 years at maximum) that mortality might seriously bias empirical results. To align the way in which simulation outcomes were recorded with the way in which the data for the ESS was collected, we took a census from the agent population twice in every simulation run (i.e. at the end of the simulation years 2010 and 2012) and pooled the data from these time points. Given the stochastic nature of our model, all the outcomes that we report below are averages over 50 independent simulation runs per experimental condition. S2 Appendix provides a detailed description of how each simulation run was initialized and also outlines the different simulation steps that take place within a run.

Our main interest was in how variation in partner preferences and the structuring effect of the educational system might affect patterns of EAM. This means that our focus was on variation in the parameters $w_{s}^{m}$, $w_{s}^{f}$, $w_{y}^{m}$, $w_{y}^{f}$, $w_{a}^{m}$, $w_{a}^{f}$, and *δ*. We fixed the remaining model parameters in the following way. First, agent populations consisted of 250 male and 250 female agents. Second, male and female agents became equally committed to their partners over time (*β* ^*m*^ = *β* ^*f*^ = .015). The values of *β* ^*m*^ and *β* ^*f*^ are based on the empirical finding that the likelihood of divorce approaches a minimum after about 25 years of marriage [cf. 57]. Choosing *β* = .015 incorporates this insight so that agents’ inclination to actively seek out alternatives to their current partner decreases concavely with the length of their current relationship and approaches 0 after about 25 to 30 years of relationship. Third, female agents experienced stronger age pressures than male agents (*σ* ^*m*^ = .0015, *σ* ^*f*^ = .0030), due to the double standard of aging. We chose the exact values of *σ* ^*m*^ and *σ* ^*f*^ based on explorative experiments that suggested that this parameterization tends to generate realistic distributions of age at first marriage among male and female agents. Fourth, we emulated the convex increase in the probability of death, which is typical for human populations, by setting *d* ^*m*^ = *d* ^*f*^ = .1 and $w_{d}^{m}=w_{d}^{f}=6$. With this parameterization, the probability that an agent dies in a given simulation year is 10% at the age of 80. Fifth, in line with our theoretical arguments, for male agents the ideal age of female partners is 24 (*α*_*i*_ = 240), whereas female agents prefer partners who are about 2.5 years older than themselves (*α*_*i*_ = *a*_*i*_ + 25). Finally, agents enter the marriage market at the age of 16; the ages at which agents make the transition from one educational level to the next, or leave the educational system, are set as described in the section ‘Agent Characteristics’ above.

### Explaining observed changes in EAM

Agent-based computational models typically leave researchers with a large degree of freedom in selecting parameter values and different parameter combinations might yield different outcomes. We therefore conducted systematic sensitivity analyses to learn about the models’ behaviour [58]. Subsequently, we compared model outcomes with data from the ESS to find the conditions under which the model is best able to explain the observed link between the gender-specific distribution of educational attainment and EAM. To avoid over-fitting the model to the idiosyncrasies of a specific country [59], we determined the conditions under which the model is *on average* best able to explain observed patterns across countries. Furthermore, to be able to validate the model with data not used in the fitting process, we split the sample and calibrated the model based on a subsample of 5 countries (Belgium, France, German, Spain, and Portugal). Subsequently, we applied the same parameterization to the full sample of 12 countries. In this section, we discuss the outcome of this last step of the analysis. We provide a detailed description of the analysis in S3 Appendix.

Note that in our calibration analysis, we required that model outcomes fit the data well in terms of both the shares of different couple types across cohorts and the average age difference within couples. We also included age differences because in most Western societies age homogamy tends to be high, although male spouses tend to be somewhat older than their female partners [37]. Given that the gender-specific distribution of educational attainment has changed over recent cohorts, age differences within couples are an important boundary condition under which our model should be able to explain observed patterns of EAM.

Based on our theoretical arguments, we expected that a model (1) in which the structuring effect of the educational system makes it more likely that agents with the same school enrolment status meet (i.e. *δ* > .5), (2) in which both men and women prefer similarly educated partners (i.e. $w_{s}^{m}>0$ and $w_{s}^{f}>0$), (3) in which both men and women prefer partners with high earnings prospects (i.e. $w_{y}^{m}>0$ and $w_{y}^{f}>0$), and (4) in which both men and women care about the age of their partners ($w_{a}^{m}>0$ and $w_{a}^{f}>0$) would generate outcomes that are similar to observed patterns of EAM.

**Fig 3:**
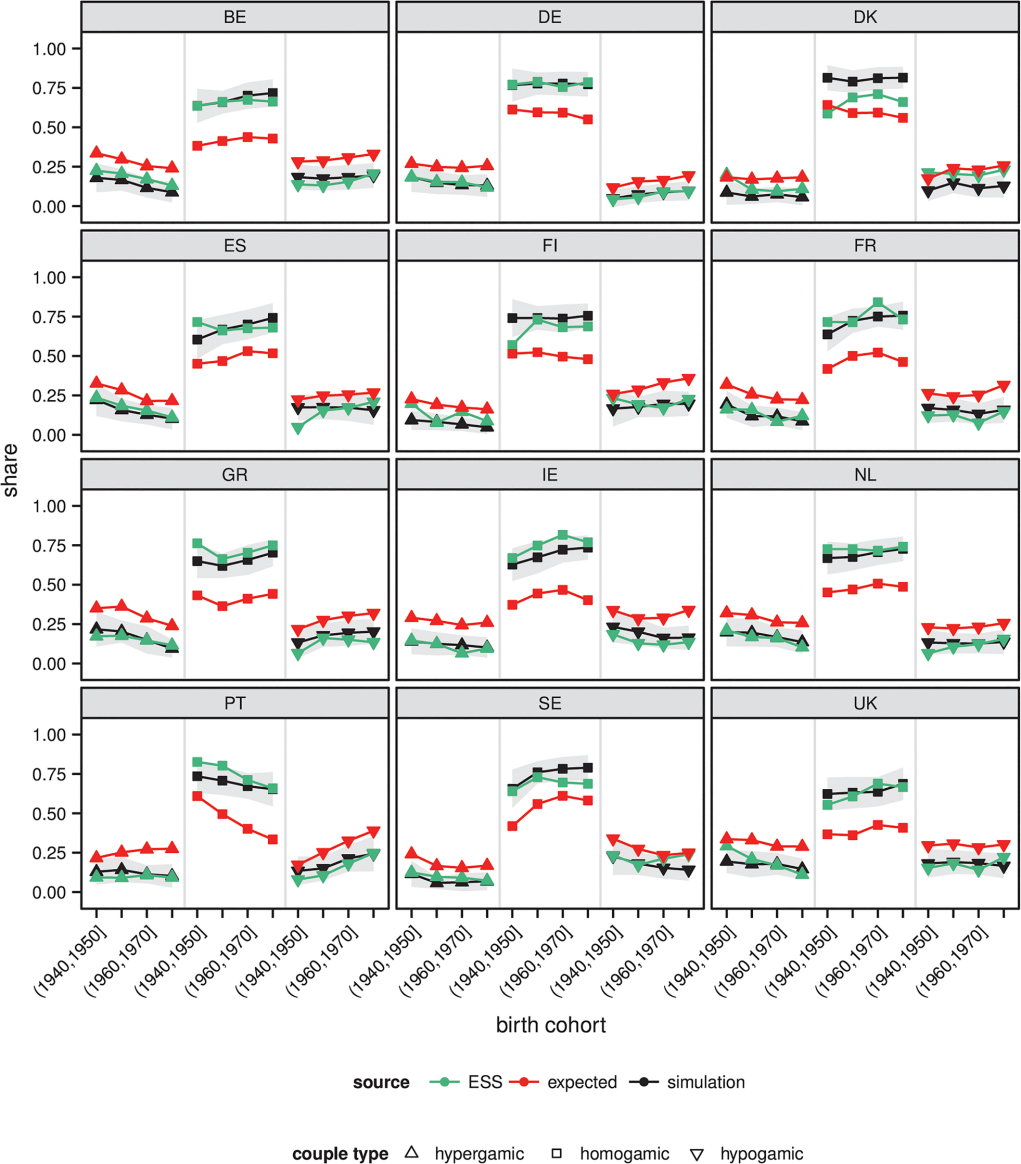
Comparison of shares of couple types in simulation outcomes and the ESS. The simulation outcomes are based on the calibrated simulation model. Confidence bands show +/- 1 standard deviation around the mean of the simulation outcomes based on 50 independent simulation runs. Weighting has been applied to the ESS data. Expected values are calculated based on the IIASA/VID data that we used for generating model inputs (see S1 Appendix for details). The calculation is based on the simplifying assumption that all men/women in a given ten-year birth cohort would marry somebody who was born in the same cohort. Based on this simplifying assumption, if marriage would occur completely at random the expected shares of hypergamic couples can be calculated as $p_{m}^{2}p_{f}^{1}+p_{m}^{3}p_{f}^{1}+p_{m}^{3}p_{f}^{2}$, the expected shares of homogamic couples can be calculated as $p_{m}^{1}p_{f}^{1}+p_{m}^{2}p_{f}^{2}+p_{m}^{3}p_{f}^{3}$, and the expected shares of hypogamic couples can be calculated as $p_{f}^{2}p_{m}^{1}+p_{f}^{3}p_{m}^{1}+p_{f}^{3}p_{m}^{2}$, where $p_{f}^{s}$ and $p_{m}^{s}$ refer to the shares of women and men in the ordered educational categories (*s*) 1 = ‘no education’/ ‘primary education’, 2 = ‘secondary education’, and 3 = ‘tertiary education’.

In line with our expectations, the calibration analysis suggested that across the subsample of 5 countries, the parameterization *δ* = .9, $w_{s}^{m}=.934$, $w_{s}^{f}=.385$, $w_{y}^{m}=1.025$, $w_{y}^{f}=1.201$, $w_{a}^{m}=5.009$ and $w_{a}^{f}=10.833$ generated outcomes that were in good accordance with the observed data. Fig 3 shows the result of applying this parameterization to all 12 countries in the sample. We also show reference values that illustrate the shares of couple types that we would expect to observe if we would assume that all members of a given ten-year birth cohort marry somebody from their own cohort, and if we would assume that within cohorts marriage occurs completely at random. The values thus roughly indicate the outcomes that we might expect to observe if the structure of the marriage market would be the only factor that affects patterns of EAM, while taking the empirically high level of age homogamy into account. We have calculated these reference values based on the IIASA/VID data.

The results suggest that the model is generally able to generate outcomes that are close to the shares of couple types observed in reality, although small deviations exist. To illustrate this, consider the results for Portugal. The observed data show that across the four cohorts considered here, there has been a steady decline in the share of educationally homogamic married couples, while there has been an increase in hypogamic couples. The same trend can be observed in the simulation outcomes. The only clear exception in terms of model fit is Denmark, in which the simulation model generates levels of homogamy/heterogamy that are consistently higher/lower than observed levels.

If we compare our simulation outcomes with the expected values, it becomes clear that *changes* in the shares of different couple types across cohorts are mostly affected by changes in the structure of the marriage market, while the processes that our model describes strongly affect the *level* of homogamy and heterogamy. To illustrate this, consider the case of Greece. Based on the composition of the marriage market, we might have expected a decrease in the share of hypergamic couples and an increase in homogamic couples similar to the changes observed in the ESS data. However, across cohorts we might have expected generally higher levels of hypergamy and hypogamy, and much lower levels of homogamy, than observed in the ESS data. Once we consider the process that our model builds upon, the shares of different couple types are much closer to the observed data.

**Fig 4:**
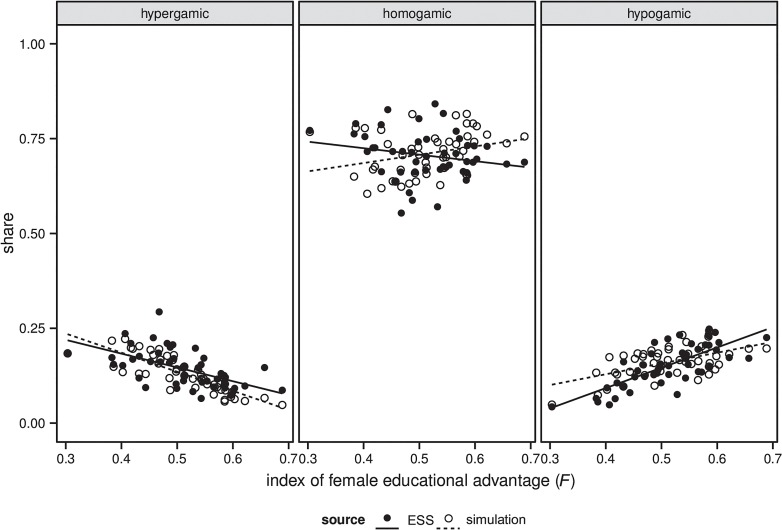
Comparison of simulated and observed association between index of female educational advantage (*F*) and EAM. The simulation outcomes are based on the calibrated model. Weighting has been applied to the ESS data. Data for calculating the index of female educational advantage (*F*) come from the IIASA/VID reconstructions/projections.

Fig 4 provides a different view on model fit. It compares the association between the index of female educational advantage (*F*) and EAM that our model generates with the association observed in the ESS data. The figure suggests that our model generates both a decrease in hypergamic couples and an increase in hypogamic couples at higher levels of female educational advantage, as observed in the ESS data. Similarly, in both the observed data and the simulation outcomes there is no strong association between female educational advantage and shares of homogamic couples, although the weak association that exists is positive in our simulation outcomes, whereas it is negative in the observed data. Table 3 additionally illustrates that also the average shares of couple types across countries and cohorts that our model generates are in good accordance with the observed data.

**Table 3:** Average shares of couple types in the ESS and in the outcomes of different versions of the simulation model.

Source	Hypergamic couples		Homogamic couples		Hypogamic couples	
	Mean	SD	Mean	SD	Mean	SD
ESS	0.14	0.05	0.71	0.06	0.15	0.06
Model: Full	0.13	0.05	0.71	0.06	0.16	0.04
Model: $w_{s}^{m}=w_{s}^{f}=0$	0.18	0.05	0.60	0.07	0.22	0.05
Model: *δ* = .5	0.16	0.05	0.64	0.07	0.21	0.05
Model: $w_{a}^{m}=w_{a}^{f}=0$	0.12	0.04	0.75	0.05	0.13	0.03
Model: $w_{y}^{m}=w_{y}^{f}=0$	0.09	0.04	0.79	0.04	0.12	0.04

The Full Model employs the parameterization obtained from our calibration analysis. The other models use the same parameterization, except for those parameters shown. Deviations from 1 in the sum of a given row are due to rounding.

Taken together, our results demonstrate that the principles of mate search that we have implemented in our ABC model can explain the observed link between changes in educational attainment and EAM. We assess next how much, according to our model, each of these principles might have contributed to this link.

### The contribution of different aspects of mate search

We conducted a second set of computational experiments in which we ‘turned-off’ certain aspects of the calibrated model and compared model outcomes to the observed data. More specifically, we conducted four experiments. In the first experiment we assumed that agents do not care about the educational attainment of their partners (i.e. $w_{s}^{m}=w_{s}^{f}=0$); in the second experiment we assumed that the educational system has no effect on meeting opportunities, so that agents are equally likely to meet somebody with the same or a different school enrolment status *r*_*i*_ (i.e. *δ* = .5); in the third experiment we assumed that agents do not care about the age of their partner (i.e. $w_{a}^{m}=w_{a}^{f}=0$); in the fourth experiment, we assumed that agents do not care about the earnings prospects of their partners (i.e. $w_{y}^{m}=w_{y}^{f}=0$).

**Fig 5:**
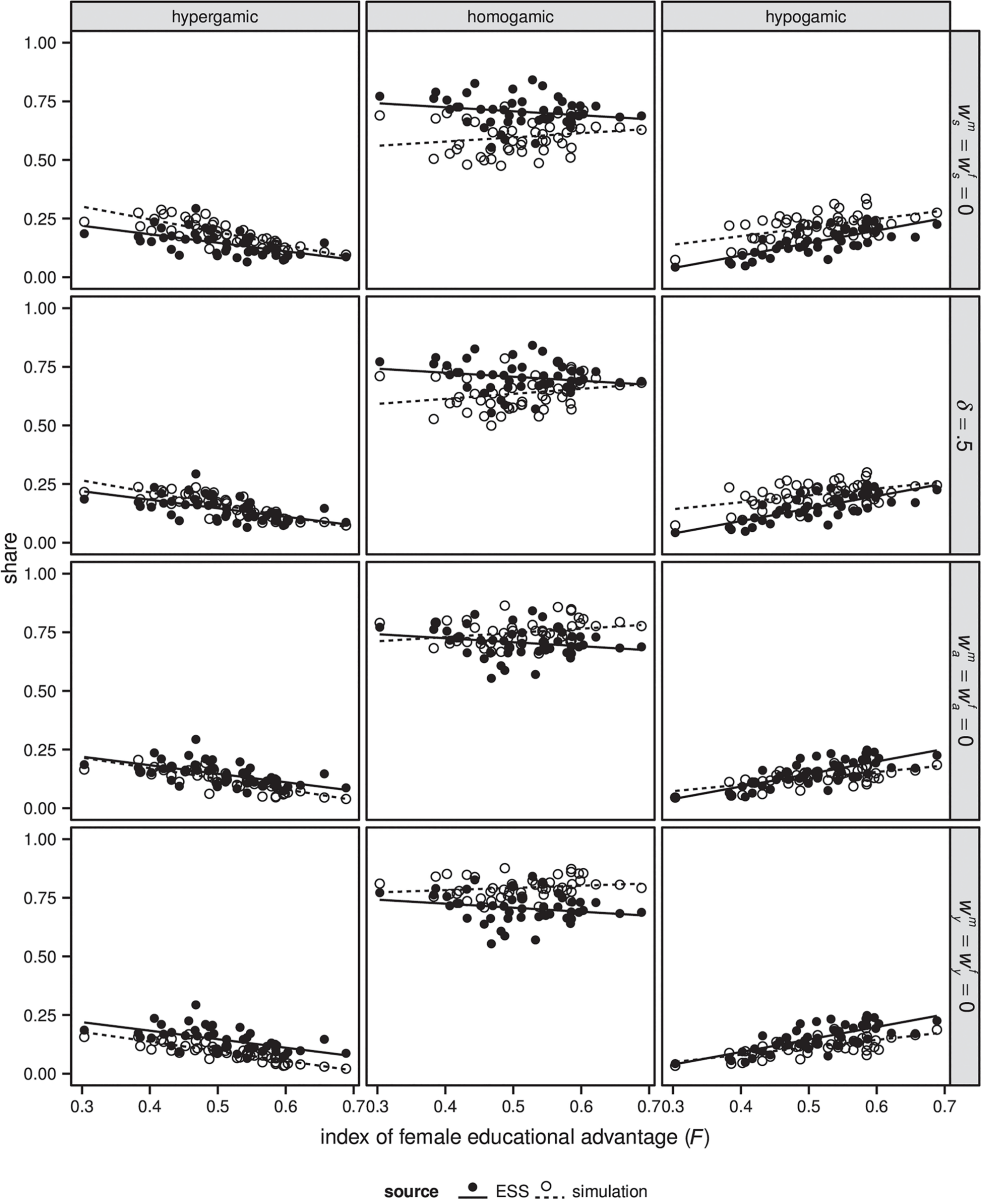
Comparison of simulated and observed association between index of female educational advantage (*F*) and EAM for different versions of the simulation model. The label of each row shows which parameters were changed compared to the calibrated version of the model. Weighting has been applied to the ESS data. Data for calculating the index of female educational advantage (*F*) come from the IIASA/VID reconstructions/projections.

Fig 5 shows the association between the index of female educational advantage (*F*) with both outcomes of different model versions and the observed data. Table 3 shows the average shares of couple types across countries and birth cohorts for the simulation outcomes and the empirical data. The results suggest that both preferences for similarly educated partners and the structuring effect of the educational system tend to increase the level of homogamy and tend to decrease the level of heterogamy. For instance, when agents do not care about the education of their partners, the average share of homogamic couples is 60%, whereas in the full model this value is 71%. Assuming that the educational system does not matter for meeting opportunities has a weaker effect. It reduces the average share of homogamic couples to 64%.

Consider next the effect that preferences for age have on model outcomes and model fit. When we compare the outcomes of this experiment with the outcomes of the other models shown in Fig 5 and in Table 3, it becomes clear that the age preferences that we consider here tend to reduce homogamy, but of all the factors that we consider they have the weakest effect on model fit. More specifically, the average share of homogamic couples in the model in which agents do not care about the age of their partner is with 75% about 4% higher than in the empirical data. By contrast, in the model that does not consider preferences for education the share is 11% lower than in the empirical data, in the model in which the educational system does not structure meeting opportunities it is 7% lower, and in the model in which preferences for earnings prospects do not matter it is 8% higher. Thus, our results suggest that in our model age preferences tend to reduce homogamy; however, assumptions about age preferences are least important for model fit, after controlling for all other mechanisms.

Consider finally the effect of preferences for earnings prospects. Our simulation outcomes suggest that if agents would not care about the earnings prospects of their partners, the level of educational homogamy would be higher (79%) than in the full model (71%). It is very likely that the high earnings prospects of highly educated agents make them attractive partners on the marriage market, even for lower educated agents. Thus, whenever highly educated agents fail to find a similarly educated partner, they should more easily be able to marry outside their own educational level. This contributes to heterogamy and reduces homogamy.

Taken together, controlling for all other aspects of the model, our results suggest that agents’ preference for similarly educated partners and for partners with high earnings prospects most strongly affect the average shares of couple types that the model generates. Agents’ preferences for partners with a particular age and the structuring effects of the educational system have comparatively weaker effects. Generally, individuals’ preferences for age are least important for the fit of model outcomes with the empirical data

## Discussion and Conclusion

We have developed an agent-based computational model that explicates some of the mechanisms that might have linked the reversal of gender inequality in higher education with observed changes in educational assortative mating across Europe. The model builds on the notion that individuals search for spouses in a marriage market and evaluate potential candidates based on preferences. The focal preferences in our model were the tendency of men and women to prefer partners with similar education and high earnings prospects. Furthermore, women tend to prefer men who are slightly older than themselves and men tend to prefer women who are in their mid-twenties. We have also incorporated the notion that the educational system can structure meeting opportunities in the population.

In our efforts to validate the model we showed that the behavioural principles that it builds upon are able to reproduce observed patterns of educational assortative mating across 12 different European countries. That is, both in the data from the 2010 and 2012 rounds of the European Social Survey and in the data from our simulation experiments, the share of married hypergamic couples decreased and the share of hypogamic couples increased in recent cohorts. These changes were correlated with changes in the gender-specific distribution of educational attainment that have occurred over the second half of the 20^th^ century. Furthermore, our results demonstrate that not only men’s and women’s tendency to prefer similarly educated partners produces high levels of homogamy, but also the structuring effects of the educational system: like marries like also because like meets like in schools and universities. By contrast, individuals’ tendency to prefer partners with high earnings prospects in particular age ranges reduces the degree of homogamy and stimulates heterogamy. Finally, our results suggest that individuals’ preferences for age are least important for the fit of model outcomes with the empirical data.

The model is generally able to generate realistic patterns of EAM, but there were also some differences between simulation outcomes and the observed data. When evaluating these differences, it is important to keep in mind that our model describes general principles of mate search and we assume that these principles operate similarly across countries and across time. The model therefore necessarily abstracts from idiosyncratic differences across countries and across time that might additionally affect patterns of assortative mating. One potentially important difference is the exact importance that men and women attach to different partner characteristics across countries. For example, existing research suggests that across Western countries women generally prefer older partners, but there seems to be some variation in precisely how much older partners should be [36]. A second potentially important difference is that such preferences might change over time. For example, among women born in older generations the preferred age difference might be larger than among members of more recent cohorts [60]. Similarly, a number of scholars have suggested that the convergence in men’s and women’s economic roles and the decline of the traditional male breadwinner model in recent decades have led to a convergence in the importance that men and women tend to attach to the earnings prospects of their partners. That is, in the past women tended to attach more importance to this characteristic in partners than men; today this importance might be more similar across the genders [13,40]. Finally, our model abstracts from historical peculiarities that might have affected observed mating patterns. Future modelling work might gain more detailed insights into the mating processes studied here, by considering such differences across countries and across time. However, our model is able to explain the link between the gender-specific distribution of educational attainment and educational assortative mating *despite* abstracting from such differences. This illustrates how general the principles of mate search are that the model builds on. Most importantly, our model shows that we can explain changes in patterns of assortative mating, without assuming that partner preferences have changed fundamentally over time. In particular, we do not need to assume that the change from the traditional pattern of hypergamy to hypogamy was based on a social preference for hypergamy that has changed over the last decades [3].

In a next step, our model can be used to develop hypotheses about future developments in assortative mating that can be tested when new data becomes available. For this, researches will need to develop scenarios of how the relative educational attainment of men and women might change in the future. The data from the International Institute for Applied Systems Analysis/Vienna Institute for Demography provide such scenarios and therefore are a good starting point for developing hypotheses. Furthermore, as indicated above, many scholars have argued that when women’s role on the labour market changes, their mate search patterns might change as well. Scenarios of future developments should take such changes, including shifting preferences, into account. The fact that our model simulates mate search over the entire life course will facilitate this process, given that researchers can easily modify or add assumptions that affect mate search in different life stages, different cohorts, and different time periods.

## Supporting Information

S1 Appendix Initializing Agent Cohorts. (DOCX)

S2 Appendix Initialization and Scheduling of Simulation Runs. (DOCX)

S3 Appendix Sensitivity Analysis and Model Calibration. (DOCX)
