# Supplementary material for: Assortative Mating and the Reversal of Gender Inequality in Education in Europe: An Agent-Based Model
Source: PLoS One. 2015 Jun 3;10(6):e0127806. doi: 10.1371/journal.pone.0127806 (PMC4454664; doi:10.1371/journal.pone.0127806)
Supplement: S1 Appendix — (DOCX) [file pone.0127806.s001.docx]

**S1 Appendix: Initializing Agent Cohorts**

We initialize the distributions of educational attainment (*s_i_*) and earnings prospects (*y_i_*) among agents born in different cohorts based on data from the IIASA/VID and the ECHP.

For initializing agent cohorts in terms of *s_i_*, we use data provided by the IIASA/VID, as described in the main part of the paper. An example helps illustrating this procedure. Assume that the simulation starts with a population born in the year 1941 in Belgium. According to the IIASA/VID data, in 1975, of those men born between 1941 and 1945 (i.e. of those who were 30-34 years old in 1975), about 22% had attained first or second stage tertiary education (i.e. educational category $s_{i}=4$ in our model). Five years later, this value had increased to about 23%. Based on this information, male agents who enter the simulation between 1941 and 1945 are assigned a *s_i_* value of 4 with probability .22. Male agents who enter the simulation about 5 years later (i.e. about 50 simulation steps later) are assigned a *s_i_* value of 4 with probability .23. We focused on the age group 30-34 years for reconstructing the educational attainment per birth cohort, given that at this age almost all individuals will have attained their highest ultimate educational degree. With this approach, we obtained input data back to cohorts born in 1936. To extend this period, we included also information about the age groups 35-39, 40-44, and 45-49 in 1970. By that, we obtained input data back to cohorts born in 1921.

For initializing agent cohorts in terms of *y_i_*, we use data from the ECHP. The ECHP provides 8 waves of survey data (collected between 1994 and 2001) with information about respondents’ education, annual income in the year prior to the survey, and recent labour history for 15 European countries. Twelve of these countries are also included in rounds 5 and 6 of the ESS. Earlier research suggests that individuals’ annual income between the mid-30s and late 40s provides a good proxy of their life-time earnings prospects [e.g., 1–4]. We therefore focused on the annual net incomes of respondents who were in the age range 36-50 in the income reference period. We excluded respondents who were self-employed in the year prior to the survey, given that from the existing research literature it is not clear how their income relates to their life-time earnings prospects. We also excluded retired respondents, given that their income might provide a biased estimate of their life-time earnings-prospects. Information about activity status was not available for The Netherlands and for Sweden. In these countries all cases were thus retained. Among the remaining respondents, we coded the income of people who were not in work with 0. After this selection, we pooled the data from the different waves into one dataset.

Based on the foregoing selection, we operationalized respondents’ earnings prospects as their income relatively to the income of the top income decile (i.e. the lower boundary of the tenth income decile in the observed data) of members of their own 5-year birth cohort, expressed in five categories (1 = less than 20% of the income of the top earning category, 2 = 20% or more and less than 40%, …, 5 = 80% or more). Focusing on relative income has the advantage that we do not need to explicitly model changes in nominal income over time due to inflation and other economic developments [5].

Based on the resulting categorization, we were able to calculated the share of men and women with tertiary (i.e. educational category $s_{i}=4$ in our model) or lower than tertiary education (i.e. educational categories$s_{i}=1$, $s_{i}=2$, and $s_{i}=3$ in our model) who fell into each of the five earnings prospects categories in the 5-year cohorts born between (1945,1950] and (1960,1965]. We decided to distinguish only between these two educational levels to avoid that low cell counts in the data for some combinations of gender, birth cohort, and detailed educational attainment aggravate effects of sampling errors. We approximated the distributions of earnings prospects among cohorts born before 1946 by using the averages of the cohorts born in (1945,1950] and (1950,1955]; we approximated the distributions of earnings prospects among cohorts born after 1966 using the averages of the cohorts born in (1955,1960] and (1960,1965].[[1]](#footnote-1)

To illustrate how we paired the data derived from the ECHP with the data from the IIASA/VID, consider again the example of Belgium. Assume a male agent born in 1941, who will ultimately attain a tertiary degree (i.e.$s_{i}=4$). According to the data derived from the ECHP, of those men born in 1941 who attained a tertiary degree, 63% had annual earnings of 80% or more of the lower boundary of the top earning decile among the members of their own 5-year birth cohort. Thus, for a male agent who is born in 1941 and who is assigned an ultimate educational level of$s_{i}=4$, there is a probability of .63 that it will be assigned the highest earning prospect category$y_{i}=5$.

**References**

1. Blomquist NS. A comparison of distributions of annual and lifetime income: Sweden around 1970. Rev Income Wealth. 1981;27(3):243–64.

2. Klevmarken NA. On the stability of age-earnings profiles. Scand J Econ. 1982;84(4):531–54.

3. Björklund A. A comparison between actual distributions of annual and lifetime income: Sweden 1951-89. Rev Income Wealth. 1993;39(4):377–86.

4. Haider S, Solon G. Life-cycle variation in the association between current and lifetime earnings. Am Econ Rev. 2006;96(4):1308–20.

5. Bosworth B, Burtless G, Steuerle E. Lifetime earnings patterns, the distribution of future social security benefits, and the impact of pension reform. Soc Secur Bull. 2000;63(4):74–98.

1. Note that the ECHP has been replaced by the European Union Statistics on Income and Living Conditions (EU-SILC) in 2003. The EU-SILC covers a larger number of countries than the ECHP. However, we decided to use the ECHP, given that the ECHP data better cover the earlier cohorts in our analysis, due to the earlier time of data collection compared to the EU-SILC data. [↑](#footnote-ref-1)
