# Supplementary material for: Assortative Mating and the Reversal of Gender Inequality in Education in Europe: An Agent-Based Model
Source: PLoS One. 2015 Jun 3;10(6):e0127806. doi: 10.1371/journal.pone.0127806 (PMC4454664; doi:10.1371/journal.pone.0127806)
Supplement: S2 Appendix — (DOCX) [file pone.0127806.s002.docx]

**S2 Appendix:** **Initialization and Scheduling of Simulation runs**

Each simulation run starts with initializing the first agent cohort from scratch. Among these agents, there cannot be any relations or marriages that might have developed through the mate search behaviour that our model specifies. Additionally, the age structure in this population cannot have developed through age-contingent mortality rates. To produce plausible starting conditions, we initialized each simulation run with a burn-in phase. This phase begins with creating a number male and female agents, for which there is a 30% probability that they are up to 19 years old, a 35% probability that they are 20 to 39 years old, a 25% probability that they are between 40 and 59 years old, and a 10% probability that they are 60 to 79 years old. To initialize agents who are born in the burn-in phase in terms of their ultimate educational attainment (*s_i_*) and earnings prospects (*y_i_*), we use data for the cohort (1920;1925], which is the first cohort for which we have input data. At the beginning of the burn-in phase all agents have the relationship status (*l_i_*) single, and their school enrolment status (*r_i_*) is set according to their age and their ultimate educational attainment. These agents then interact, reproduce, and die over a period of 80 years (i.e. 800 simulation steps). We use the resulting agent population at the end of this burn-in phase as the first cohort in the actual simulation process, which starts in the simulation year 1921.

Fig. 1 provides a flow diagram of the central steps in the simulation process. The step labelled ‘Meeting and dating decisions’ consists of the following sub-steps (these steps are conducted once for each agent in the population):

1. Randomly select one agent (without replacement) and determine whether the focal agent *i* actively tries to meet another agent *j* in the current iteration.
2. If agent *i* actively tries to meet another agent *j*:
   1. Determine whether *j* will be somebody with the same school enrolment status as *i* (*r_i_* = *r_j_*), or somebody with a different school enrolment status (*r_i_* ≠ *r_j_*).
   2. If the set of agents who satisfy the criterion determined in (1) is not empty:
      1. Randomly select an agent *j* from the set of agents who satisfy the criterion determined in (1).
      2. Determine for *i* and *j* whether they want to start dating the respective other.
      3. If both agents want to date, remove possible relations with current partners, create a new relation between them, update their relationship status (*l_i_*); also update (*l_i_*) for agents who are broken up with/divorced when a new relation is formed.


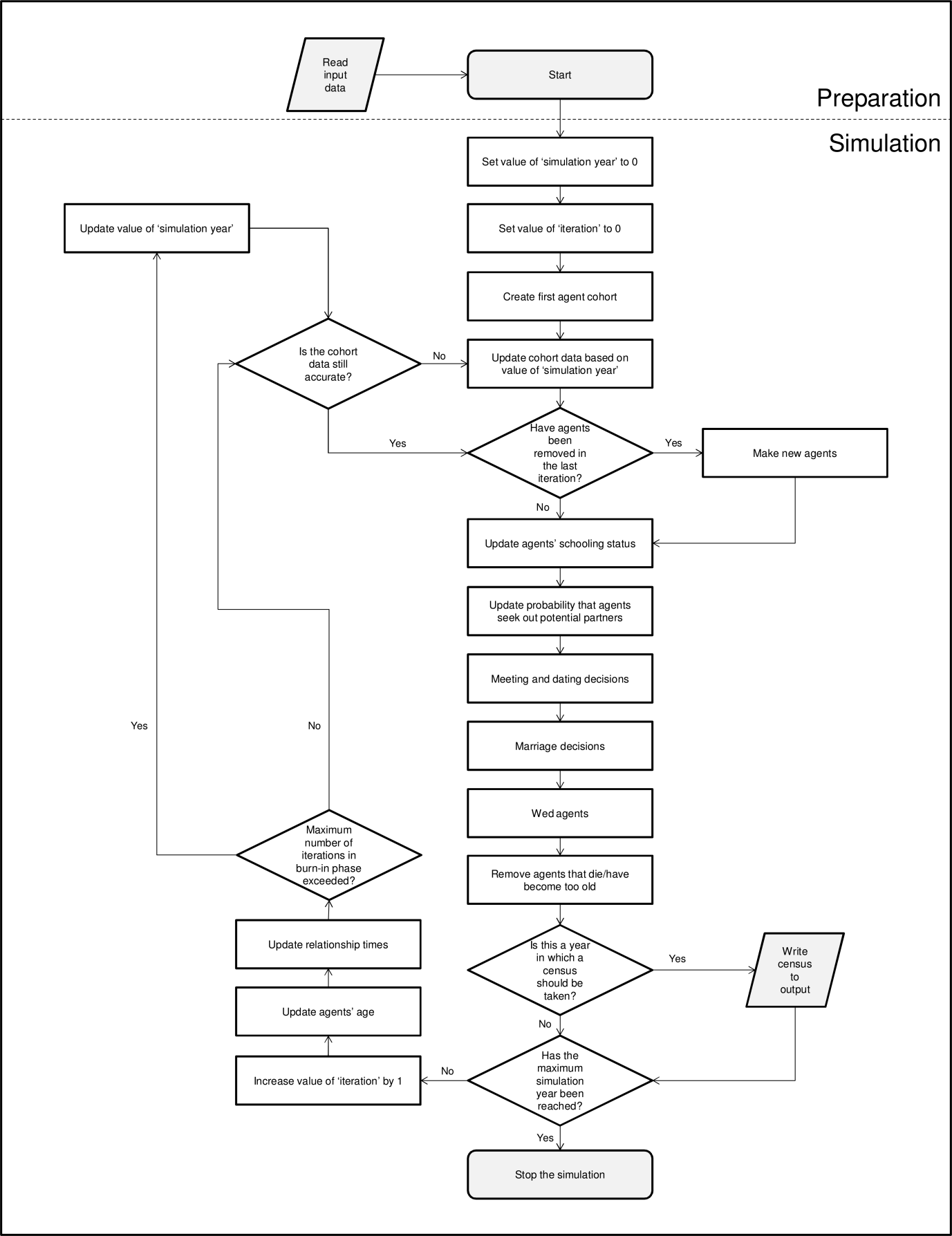


**Fig. 1.** Flow diagram of simulation process.
