# Supplementary material for: Assortative Mating and the Reversal of Gender Inequality in Education in Europe: An Agent-Based Model
Source: PLoS One. 2015 Jun 3;10(6):e0127806. doi: 10.1371/journal.pone.0127806 (PMC4454664; doi:10.1371/journal.pone.0127806)
Supplement: S3 Appendix — (DOCX) [file pone.0127806.s003.docx]

**S3 Appendix: Sensitivity Analysis and Model Calibration**

The goal of our sensitivity analysis and model calibration was to explore under which conditions the simulation model is best able to explain observed patterns of EAM. An increasing number of scholars advocate the use of principles from the theory of the design of experiments for systematically studying the behaviour of simulation models [1,2]. With this approach, we treat the simulation model as a black box and assess the relation between the parameters of the simulation model and simulation outcomes (the so-called ‘response’) with a metamodel. In most applications, a second order polynomial is sufficiently complex for studying the response surface that a simulation model generates [1]. The polynomial has the form of

$u=b_{0}+\sum_{p=1}^{X} b_{p}x_{p}+\sum_{p=1}^{X-1} \sum_{q=p+1}^{X} b_{pq}x_{p}x_{q}+\sum_{p=1}^{X} b_{pp}x_{p}^{2}+\varepsilon$, (1)

where *u* is the focal simulation outcome, *b_0_* is an intercept, *X* is the number of parameters in the simulation model, *x_p_*/*x_q_* and *b_p_*/*b_q_* refer to specific parameters of the simulation model and associated coefficients, and *ε* is a residual error term. The model defined in Eq. (1) can be estimated with ordinary least square regression and the resulting coefficients provide insights into how simulation outcomes change on average when the value of a given parameter of the simulation model is increased by one unit (controlling for all other parameters). It also provides insights into possible non-linear effects and into the possible interplay between parameters in generating simulation outcomes.

The simulation outcome of interest in our analysis (i.e. *u* in Eq. (1)) was the fit of the simulation outcomes with the observed data in terms of the shares of different couple types in the four birth cohorts (1940 – 1950], (1950 – 1960], (1960 – 1970]), and (1970 – 1980]) and in terms of the average age difference within couples (his age - her age) in these cohorts. We first calculated the deviation of model outcomes from the observed data (*dev*) in a given experimental condition as

$dev=\left(\sum_{n=1}^{4} \left|hyper_{^{n}}^{obs}-hyper_{^{n}}^{sim}\right|*\sum_{n=1}^{4} \left|\text{homo}_{^{n}}^{obs}-\text{homo}_{^{n}}^{sim}\right|*\sum_{n=1}^{4} \left|\text{hypo}_{^{n}}^{obs}-\text{hypo}_{^{n}}^{sim}\right|*\sum_{n=1}^{4} \left|\text{agediff}_{^{n}}^{obs}-\text{agediff}_{^{n}}^{sim}\right|\right)^{1/4}$, (2)

where *n* indexes the four birth cohorts under consideration, *hyper*, *homo*, and *hypo*, refer to the shares of hypergamic, homogamic, and hypogamic couples, *agediff* refers to the average age difference within couples, and *obs* and *sim* refer to empirically observed data and data obtained from the simulation model. Subsequently, we calculated the fit of simulation outcomes with the observed data as$fit=-log\left(dev\right)$. We applied a log transformation, given that *dev* tended to be right skewed. Note that Eq. (2) is based on standard recommendations for calculating model fit based on multiple dimensions that need to be optimized [3].

Using a metamodel for assessing the conditions under which *fit* is maximized has the advantage that the behaviour of the simulation model can be assessed from a comparatively small set of simulation runs. This is particularly attractive if the model is computationally expensive or has many parameters whose interactions need to be considered. Several experimental designs make the efficient estimation of second order polynomials possible. The design that is most commonly recommended is the Central Composite Design (CCD) [3]. Our focus on the effects of the model parameters$w_{s}^{m}$,$w_{s}^{f}$,$w_{y}^{m}$,$w_{y}^{f}$,$w_{a}^{m}$,$w_{a}^{f}$, and *δ* implies that there are 7 experimental factors. A full CCD with 7 experimental factors and 5 levels per factor requires 150 design points/experimental conditions. It includes 8 centre runs for calculating the lack of fit statistic that enables us to assess whether the metamodel is an appropriate representation of the relation between model parameters and model outcomes [see 3 for details on how the number of required design points is calcualted].[[1]](#footnote-1) Given the stochastic nature of our model, we conducted 50 independent simulation runs at each design point and average outcomes over these runs before calculating *fit*.

Table 1 shows the factor levels in original and coded units. We provide the full experimental schedule in the supplementary material that accompanies the main part of the article. For the lower boundary of the experimental region, we used the value 0 as the lower operational boundary of all model parameters. We determined the upper boundaries of$w_{s}^{m}=w_{s}^{f}=w_{y}^{m}=w_{y}^{f}=2$, and $w_{a}^{m}=w_{a}^{f}=20$ through exploratory experimentation, which suggested that increasing parameters beyond these values did do not change outcomes much anymore. The upper operational boundary of *δ* is 1. Given these boundaries, the centre of the CCD was at$w_{s}^{m}=w_{s}^{f}=w_{y}^{m}=w_{y}^{f}=1$,$w_{a}^{m}=w_{a}^{f}=10$, and $\delta=.5$. To avoid over-fitting the model to the peculiarities of a specific country, we took a subsample of five countries (Belgium, France, German, Spain, and Portugal) and conducted the same experiment for each of them. Subsequently, we pooled the results from all countries, which generated 750 observations in total.

A formal test for homogeneity of the error variance [4] suggested that there was some heteroscedasticity in the residuals of the ordinary least square model. Thus, as commonly recommended in the literature on simulation modelling, we employed weighted least square regression to avoid that this heteroscedasticity might bias our test statistics [cf. 5]. Table 2 reports the estimates of the response surface of *fit* and Table 3 reports the analysis of variance of the response surface and the lack of fit statistic. The lack of fit statistic suggests that the metamodel provides a good approximation of the response surface and the estimated coefficients suggest that the shape of the surface is most strongly affected by the parameters$w_{s}^{m}$,$w_{s}^{f}$, and *δ*, as well as by all corresponding quadratic terms and interaction terms between them. We also conducted a canonical analysis to find the conditions under which predicted model fit is highest [3]. This analysis suggested that there was a stationary point on the response surface at $w_{s}^{m}=1.107$,$w_{s}^{f}=.669$, $w_{y}^{m}=1.236$, $w_{y}^{f}=1.541$,$w_{a}^{m}=4.955$, $w_{a}^{f}=10.836$ and $\delta=.75$. The predicted stationary point was a saddle point, as indicated by mixed signs of the eigenvalues associated with each model parameter. That is, the predicted stationary point was neither a minimum nor a maximum. In such a situation, the predicted value of *fit* could be improved by adjusting parameters in one of two opposing directions. Table 4 provides insights into these directions by reporting the results of a ridge analysis around the predicted stationary point [3].[[2]](#footnote-2)

From a technical standpoint, based on the estimated coefficients of the metamodel we might have maximized *fit* by setting *δ* to 0 or 1 and by choosing all other parameters accordingly. This would imply that while in school, agents never ($\delta=0$)/only ($\delta=1$) meet other agents who are enrolled in the same educational level. It is well known that adolescents spend most of their time at school and that interactions with other adolescents who are attending the same educational level tend to dominate their daily interactions [6]. However, it is unlikely that these interactions make up for 100% of their interactions. We therefore chose a value of $\delta=.9$, which implies strong structuring effects of the educational system, but leaves some room for interactions among agents who are not enrolled in the same educational level. Accordingly, we choose the other model parameters as $w_{s}^{m}=.934$,$w_{s}^{f}=.385$, $w_{y}^{m}=1.025$, $w_{y}^{f}=1.201$, $w_{a}^{m}=5.009$ and $w_{a}^{f}=10.833$.

**References**

1. Kleijnen JPC. An overview of the design and analysis of simulation experiments for sensitivity analysis. Eur J Oper Res. 2005;164:287–300.

2. Lorscheid I, Heine B-O, Meyer M. Opening the “black box” of simulations: increased transparency and effective communication through the systematic design of experiments. Comput Math Organ Theory. 2012;18(1):22–62.

3. Myers RH, Montgomery DC. Response surface methodology: process and product optimization using designed experiments. New York: John Wiley & Sons, Inc.; 1995.

4. Breusch TS, Pagan AR. A simple test for heteroscedasticity and random coefficient variation. Econometrica. 1979;47(5):1287–94.

5. Tunali S, Batzman I. Dealing with the least squares regression assumptions in simulation metamodeling. Comput Ind Eng. 2000;38(2):307–20.

6. Coleman JS. The adolescent society. the social life of the teenager and its impact on education. New York: Free Press; 1961.

**Tables**

**Table 1.** Model parameters in coded and original units.

| **Coded units** | **Original units** | | |
| --- | --- | --- | --- |
|  |  |  | *δ* |
| -1 | 0 | 0 | 0 |
| -0.297 | 0.703 | 7.027 | 0.351 |
| 0 | 1 | 10 | 0.5 |
| 0.297 | 1.297 | 12.973 | 0.649 |
| 1 | 2 | 20 | 1 |

**Table 2.** Estimates of second order polynomial predicting *fit*.

| **Parameters** | ***b*** | **p** |
| --- | --- | --- |
| intercept | -1.75 | * |
| $w_{s}^{m}$ | 1.47 | ** |
| $w_{s}^{f}$ | 1.49 | ** |
| *δ* | 5.14 | ** |
| $w_{a}^{m}$† | 0.10 |  |
| $w_{a}^{f}$† | -0.01 |  |
| $w_{y}^{m}$ | 0.36 |  |
| $w_{y}^{f}$ | -0.33 |  |
| $w_{s}^{m}$*$w_{s}^{f}$ | -0.37 | ** |
| $w_{s}^{m}$* *δ* | -1.22 | ** |
| $w_{s}^{m}$*$w_{a}^{m}$† | 0.01 |  |
| $w_{s}^{m}$*$w_{a}^{f}$† | 0.12 |  |
| $w_{s}^{m}$*$w_{y}^{m}$ | 0.07 |  |
| $w_{s}^{m}$*$w_{y}^{f}$ | 0.18 |  |
| $w_{s}^{f}$* *δ* | -1.62 | ** |
| $w_{s}^{f}$*$w_{a}^{m}$† | 0.10 |  |
| $w_{s}^{f}$ *$w_{a}^{f}$† | 0.23 |  |
| $w_{s}^{f}$*$w_{y}^{m}$ | 0.06 |  |
| $w_{s}^{f}$*$w_{y}^{f}$ | 0.15 |  |
| *δ**$w_{a}^{m}$† | 0.34 |  |
| *δ**$w_{a}^{f}$† | 0.68 | * |
| *δ**$w_{y}^{m}$ | -0.38 |  |
| *δ**$w_{y}^{f}$ | 0.16 |  |
| $w_{a}^{m}$*$w_{a}^{f}$† | 0.00 |  |
| $w_{a}^{m}$*$w_{y}^{m}$† | -0.18 |  |
| $w_{a}^{m}$*$w_{y}^{f}$† | -0.09 |  |
| $w_{a}^{f}$*$w_{y}^{m}$† | 0.22 |  |
| $w_{a}^{f}$*$w_{y}^{f}$† | -0.07 |  |
| $w_{y}^{m}$*$w_{y}^{f}$ | 0.06 |  |
| $w_{s}^{m}$² | -0.36 | ** |
| $w_{s}^{f}$² | -0.34 | ** |
| *δ*² | -2.26 | ** |
| $w_{a}^{m}$²† | -0.01 |  |
| $w_{a}^{f}$²† | -0.04 | ** |
| $w_{y}^{m}$² | -0.17 |  |
| $w_{y}^{f}$² | -0.01 |  |

† coefficient multiplied by 10 to facilitate legibility

** p < .01, * p < .05

**Table 3.** Analysis of variance of second order polynomial predicting *fit*.

| **Source** | **DF** | **Sum of squares** | **Mean square** | **F-value** | **p** |
| --- | --- | --- | --- | --- | --- |
| linear | 7 | 124.12 | 17.73 | 16.92 | ** |
| interaction | 21 | 74.83 | 3.56 | 3.40 | ** |
| quadratic | 7 | 41.74 | 5.96 | 5.69 | ** |
| total error | 714 | 748.28 | 1.05 |  |  |
| lack of fit | 107 | 40.03 | 0.37 | 0.32 |  |
| pure error | 607 | 708.25 | 1.17 |  |  |

** p < .01, * p < .05

**Table 4.** Ridge analysis around estimated stationary point of second order polynomial predicting *fit*.

| **Dist. from stat. point** | $w_{s}^{m}$ | $w_{s}^{f}$ | *δ* | $w_{a}^{m}$ | $w_{a}^{f}$ | $w_{y}^{m}$ | $w_{y}^{f}$ | **Pred. value of *fit*** |
| --- | --- | --- | --- | --- | --- | --- | --- | --- |
| -2 | 0.473 | -0.376 | 1.299 | 5.156 | 10.825 | 0.463 | 0.290 | 1.792 |
| -1.5 | 0.631 | -0.115 | 1.162 | 5.105 | 10.828 | 0.656 | 0.603 | 1.654 |
| -1 | 0.790 | 0.146 | 1.024 | 5.055 | 10.831 | 0.849 | 0.916 | 1.555 |
| -0.5 | 0.948 | 0.408 | 0.887 | 5.005 | 10.833 | 1.043 | 1.228 | 1.495 |
| 0 | 1.107 | 0.669 | 0.750 | 4.955 | 10.836 | 1.236 | 1.541 | 1.476 |
| 0.5 | 1.265 | 0.931 | 0.613 | 4.904 | 10.839 | 1.429 | 1.854 | 1.495 |
| 1 | 1.424 | 1.192 | 0.476 | 4.854 | 10.842 | 1.622 | 2.167 | 1.555 |
| 1.5 | 1.582 | 1.453 | 0.339 | 4.804 | 10.845 | 1.816 | 2.480 | 1.654 |
| 2 | 1.740 | 1.715 | 0.202 | 4.753 | 10.848 | 2.009 | 2.793 | 1.792 |

1. We used an *alpha* value of 3.364 to make the CCD rotatable [3]. Note that *alpha* refers here to the value that needs to be chosen for selecting the distance of the design points from the center of the CCD (referred to as *α* in standard text books on CCDs) and *not* to the age-pressure factor *α* in our simulation model. We required that the CCD is inscribed to avoid that design points fall outside the operational region of the model. [↑](#footnote-ref-1)
2. In the original ordinary least square analysis there was one data point for which the standardized residual in the regression model was larger than 3.5. Including/removing this data point from the analysis affected the estimates for the stationary point of the response surface. Instead of simply removing this data point from the analysis, we re-ran the simulation model for the corresponding parameter combination, to take the possibility into account that this deviation might be due to chance. After replacing the results for this parameter combination obtained in the original experiment with the new results, the estimation of the response surface was robust to the inclusion/exclusion to this data point. [↑](#footnote-ref-2)
